# The cost-effectiveness of school-based interventions for chronic diseases: a systematic review

**DOI:** 10.1186/s12962-024-00511-w

**Published:** 2024-04-11

**Authors:** George Lin, Kalin Werner, Ada Alqunaiebet, Mariam M. Hamza, Norah Alkanhal, Reem F. Alsukait, Amaal Alruwaily, Severin Rakic, Volkan Cetinkaya, Christopher H. Herbst, Tracy Kuo Lin

**Affiliations:** 1 Department of Psychiatry and Behavioral Sciences, Stanford University, Stanford, CA USA; 2 Institute for Health & Aging, Department of Social and Behavioral Sciences, University of California, San Francisco, CA USA; 3 Saudi Public Health Authority, Riyadh, KSA Saudi Arabia; 4 Health, Nutrition and Population Global Practice, The World Bank, Washington, DC USA; 5 College of Applied Medical Sciences, King Saud University, Riyadh, Saudi Arabia

**Keywords:** Cost-effectiveness, School, Chronic diseases, Non-communicable diseases, Systematic review

## Abstract

**Background:**

Chronic diseases, or non-communicable diseases (NCD), are conditions of long duration and often influenced and contributed by complex interactions of several variables, including genetic, physiological, environmental, and behavioral factors. These conditions contribute to death, disability, and subsequent health care costs. Primary and secondary school settings provide an opportunity to deliver relatively low cost and effective interventions to improve public health outcomes. However, there lacks systematic evidence on the cost-effectiveness of these interventions.

**Methods:**

We systematically searched four databases (PubMed/Medline, Cochrane, Embase, and Web of Science) for published studies on the cost-effectiveness of chronic-disease interventions in school settings. Studies were eligible for inclusion if they assessed interventions of any chronic or non-communicable disease, were conducted in a school setting, undertook a full cost-effectiveness analysis and were available in English, Spanish, or French.

**Results:**

Our review identified 1029 articles during our initial search of the databases, and after screening, 33 studies were included in our final analysis. The most used effectiveness outcome measures were summary effectiveness units such as quality-adjusted life years (QALYs) (22 articles; 67%) or disability-adjusted life years (DALYs) (4 articles; 12%). The most common health condition for which an intervention targets is overweight and obesity. Almost all school-based interventions were found to be cost-effective (30 articles; 81%).

**Conclusion:**

Our review found evidence to support a number of cost-effective school-based interventions targeting NCDs focused on vaccination, routine physical activity, and supplement delivery interventions. Conversely, many classroom-based cognitive behavioral therapy for mental health and certain multi-component interventions for obesity were not found to be cost-effective.

**Supplementary Information:**

The online version contains supplementary material available at 10.1186/s12962-024-00511-w.

## Background

Chronic diseases, or non-communicable diseases (NCD), are conditions of long duration. They are influenced by complex interactions of several variables, including genetic, physiological, environmental, and behavioral factors [1]. Examples of NCD include cardiovascular diseases (e.g., myocardial infarction, cerebral vascular accident), cancer, chronic respiratory diseases (e.g., chronic obstructive pulmonary disease, asthma), and diabetes mellitus. NCDs represent more than half of the global burden of disease in the context of the chronic disease epidemic [2]. These conditions contribute to death, disability, and subsequent health care costs.

An advantageous approach to reducing risk factors of NCD is by targeting modifiable behaviors. Childhood and adolescence are crucial periods and opportunities for achieving health gains. The promotion of healthy behaviors, psychoeducation, and effective early clinical interventions can develop positive behaviors that can directly benefit children and adolescents as well as be disseminated to family members, resulting in greater community-wide intervention [1].

Recognizing the criticality, the World Health Organization (WHO) launched the Global School Health Initiative in 1995 with the purpose of advocating worldwide the Health Promoting School (HPS) approach, which is characterized by the WHO as “a school constantly strengthening its capacity as a healthy setting for living, learning and working” [3]. The goal of the initiative was to advocate for the development of school health programs and increase the number of health-promoting schools with the goal of improving child, adolescent and community health through health promotion and programming within school setting. School-based interventions may be effective as it can offer multiple layers of intervention that can be broadly categorized into three categories: primary interventions which utilize universal strategies that can be applied to all students in school settings to promote knowledge and health-positive behavior; secondary interventions which utilize prevention strategies targeting students at risk for developing chronic problem behaviors; and tertiary interventions which target students who present with pervasive behavioral challenges and provide treatment through individualized services [4].

With the launch of HPS, there have been numerous studies that evaluated the effectiveness as well as cost-effectiveness of school-based interventions—defined here as programs implemented in classroom or school-settings and designed for improving the health and well-being of students—for alleviating the prevalence of NCDs [5–8]. School-based interventions may target specific pathology or problematic behaviors and improve many public health issues, such as physical health (e.g., through reducing the prevalence of overweight and obesity and smoking) [9] and mental health [10]. These programs often require interdisciplinary coordination between school personnel, administration, professional, and may coordinate with parents and the community and may involve specifically designed lectures, activities, and encouraging positive behavioral outcome. The costs of these programs are often paid for by international organizations, national, federal, local governments, or charity foundations.

Given the finite resources available for public health promotion, it is important not only to evaluate but also to compare the cost-effectiveness of these programs. Such comparison will allow decision makers to determine the most cost-effective intervention that is suitable for specific settings, informing evidence-based resource allocation and ensuring that the fundings are allocated to interventions that may maximize health benefits. To our knowledge, there is an absence of a systematic review and comparison of the cost-effectiveness of school-based interventions for ameliorating and mitigating NCD. The aim of this systematic literature review is to identify and summarize the published evidence on the cost-effectiveness of health enhancement interventions—including health promotion, prevention, and treatment—for chronic disease in school-based settings. The findings will supply policymakers with comprehensive evidence and systematic comparisons regarding cost-effective strategies to alleviate the prevalence of chronic diseases and serve to inform the development of future health behavior interventions in school settings.

## Methods

### Study design

We conducted a review following Preferred Reporting Items for Systematic Reviews and Meta-Analyses (PRISMA) reporting standards and registered with PROSPERO (CRD42022324101). We systematically searched four databases (PubMed/Medline, Cochrane, Embase, and Web of Science) for published literature on the cost-effectiveness of chronic disease interventions in school-based settings. Search strings were developed using a combination of MeSH and text word searches to cover the following concepts: (1) chronic disease (2) health promotion or awareness interventions (3) school settings (4) economic evaluations.

The full search strategy is available in Additional file 1: Table S1 and an example of terms used in our search is provided in Box 1.

## Box 1. Sample PubMed search strategy



### Eligibility criteria

Studies were included in our review if they assessed interventions of any chronic or non-communicable disease, were conducted in a school setting, undertook a full cost-effectiveness analysis (specifically cost-effectiveness analysis or cost-utility analysis) and were available in English, Spanish or French. Studies that did not include a school-based component, where the analyzed or simulated population did not focus on children or adolescents in primary or secondary school (i.e., persons under 18 years of age), or did not calculate an incremental cost-effectiveness ratio specifically for this population sub-group were excluded.

This review also focused on cost-effective and cost-utility analyses where the outcome is a health outcome measure (as opposed to a process measure). For example, for obesity-related interventions, we included studies that examined the cost per body mass index (BMI) reduced and excluded studies that evaluated the cost per minute exercised; we reasoned the per minute exercised is a process outcome that may or not translate to BMI reduction.

On the other hand, we elected to include interventions that aimed to improve intermediate health steps that are not merely process outcomes—as many of these health steps are strongly associated with health outcomes. In one case, we included human papillomavirus (HPV) vaccination due to its association with cancer prevention. In another case, we included interventions to reduce instances of bullying at school as bullying has a direct impact on mental health in general and anxiety in particular.

Duplicates were removed using Covidence review software (Veritas Health Innovation, Melbourne, Australia. Available at www.covidence.org), and secondarily hand checked by the study team. For each study, two of the three reviewers from the study team independently screened studies for eligibility, extracted data, and assessed the risk of bias. Conflicts were resolved through discussion among the reviewers and resolved by a third reviewer when necessary. Studies were first examined by title and abstract. After removing studies that did not meet the inclusion criteria, the full text of the remaining articles was screened again for eligibility. All references within included studies were checked to identify additional relevant articles. No ethical approval was required for this desk-based review.

### Risk of bias assessment

Each study was assessed for risk of bias by a single reviewer using the Consensus on Health Economic Criteria (CHEC) list [11]. The tool included 19 criteria, focused on study design, with items ranging from a clear description of competing alternatives to the appropriate measurement of costs and value of outcomes, to inform how well an article has addressed the minimum quality elements of an economic evaluation. Scores of this assessment can be found in Additional file 2: Supplementary Material Table 1. Studies that achieved lower than 80% (15 out of 19) of the CHEC list were considered to be at high risk of bias and of low quality, and therefore excluded from our review. We reason that this step is especially critical for a systematic review that focuses on cost-effectiveness analyses as studies without relevant information to provide context render the results opaque and difficult to evaluate. Details of our risk bias assessment are presented in Additional file 2: Supplementary Material Table 1.

### Data extraction and analysis

Data were extracted from included studies using a predetermined 21-item matrix that was programed into Covidence software platform. A summary of key data extracted from each study included in our review is provided in Additional file 2: Supplementary Material Table 2.

Study outcomes were first summarized in a narrative synthesis. Due to study heterogeneity and a lack of explicitly reported incremental costs and outcomes for each study, we were not able to conduct a meta-analysis of incremental net benefits. However, results that analyzed similar alternatives were compared where possible.

### Limitations

We note that publication bias may result in the tendency of an overrepresentation of studies with positive results. Despite such tendency, our review identified six studies that did not demonstrate the cost-effectiveness of the intervention of interest, as compared to a relevant comparator. There is a broad number of diseases considered under the umbrella of NCDs which leads to heterogeneity of interventions and diseases addressed in our review. Results from economic evaluations are highly dependent on model choices including structure, input parameters, assumptions, and reported outcomes, as well as the geography and context of the assessed intervention. Our review did not place geographical limitations on our search and thus the data identified in our review is not necessarily comparable. However, we find that the results are consistent and adequate for drawing broad conclusions about the cost-effectiveness of school-based chronic disease interventions.

## Results

**Fig. 1:**
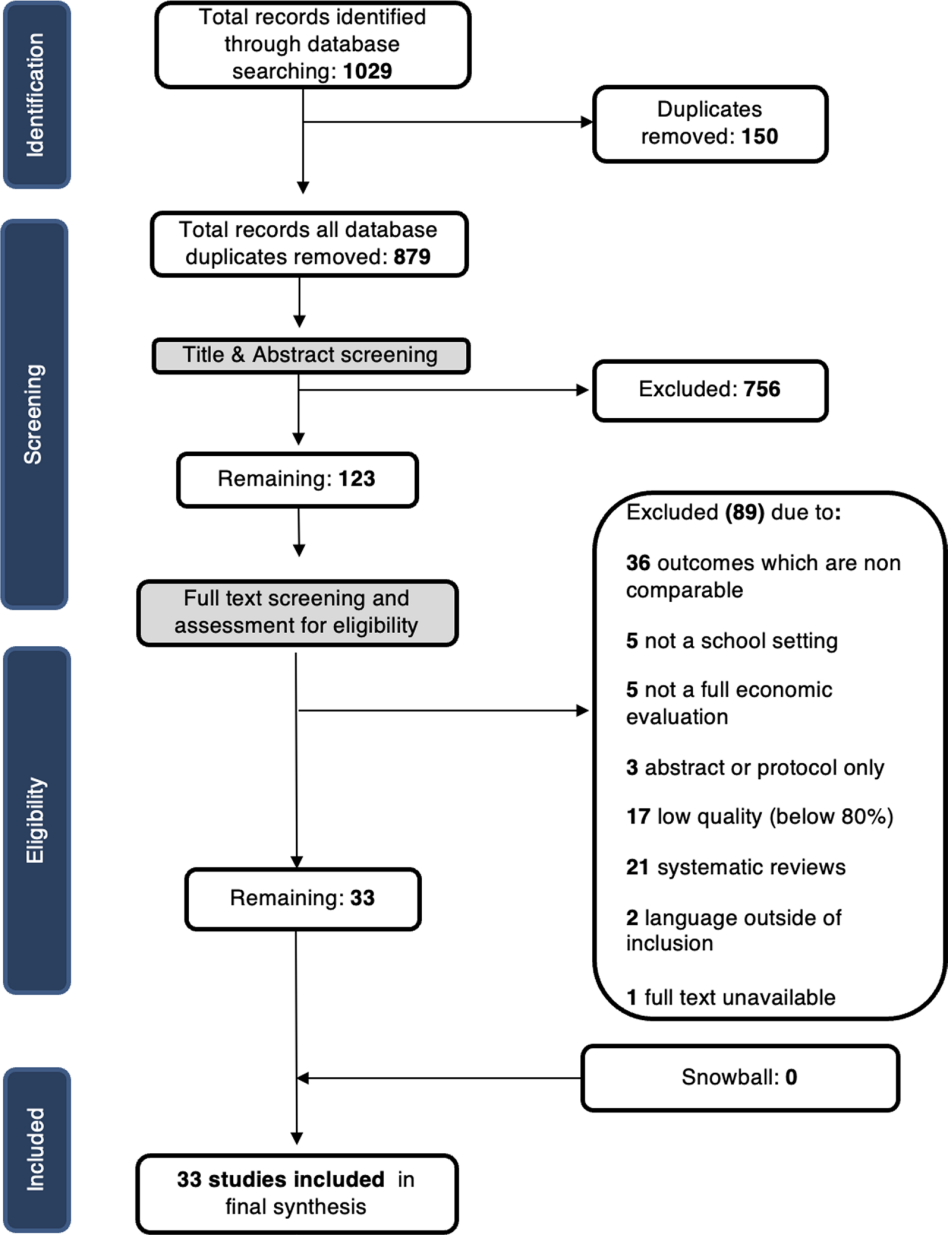
PRISMA diagram of screening results

Search results and subsequent screening are presented in Fig. 1. Initial database searches identified 1029 studies. 150 duplicates were removed leaving 879 studies for initial screening of title and abstracts. After removing 756 irrelevant studies, 123 papers were eligible for full-text review. During full-text screening articles 36 articles did not assess a comparable health outcome measure. Five studies were not conducted in a school setting and five were not full economic evaluations. Three studies were available as abstracts only, two papers were excluded due to language limitations and one article was unavailable as a complete text. Twenty-one studies were excluded based on being systematic reviews, however reviewers hand searched the references lists of these articles for additional literature. After the quality assessment, 17 studies were considered at high risk of bias and excluded. The final review included 33 studies.

### Descriptive characteristics

We extracted and discussed eight descriptive characteristics from included studies. Summaries of these charateristics are presented below and additional details are presented in Additional file 2: Supplementary Material Table 2.

#### Perspective chosen

Studies varied in the type of costs and effects considered in their analysis. A majority of studies undertook their analysis from a societal perspective (15 articles; 45%) while the remainder considered public sector (8 articles; 24%) or health care sector (7 articles; 21%) costs. A single study used both a payor perspective and a school system perspective. One study compared the analysis both from the societal perspective and public sector perspective, and one study did not report on the perspective chosen in the analysis.

#### Intervention and comparator

Personal awareness or health promotion strategies were covered in the highest frequency (n = 7), followed by multicomponent interventions (n = 6) and structured physical activity (n = 5). Other interventions for reducing NCDs included strategies such as HPV vaccination (n = 4), classroom-based cognitive behavioral therapy (n = 3), nutritional supplementation distribution (n = 2), training staff and teachers for classroom management (n = 2) and other psychoeducation interventions (n = 1).

Most studies compared interventions against no intervention (17 articles; 52%) or usual care, activities, and education (9 articles; 27%). Two studies that assessed HPV vaccination compared vaccines that varied in the number of types of HPV protection (e.g., nonavalent versus bivalent).

#### Setting and simulated population

We identified 12 studies (36%) that focused on interventions in secondary schools and 12 studies (36%) that focused on interventions in primary school settings. Three studies (9%) assessed interventions that were conducted in school-based and additional settings, such as community centers of health facilities. Seven studies (21%) did not specify the setting of their intervention. The simulated populations of analyses varied in age and reflected the setting under which the analysis was conducted.

#### Targeted diseases

Interventions targeted an array of chronic illnesses. The most common disease was obesity and/or overweight (14 articles; 42%) and the closely related issue of type 2 diabetes (2 articles; 6%). Ten studies addressed mental health (30%) including depression, anxiety, self-harm, bullying and aggression. Tobacco use was targeted in two studies, and four studies included interventions aimed to reduce HPV infections. A single study addressed cardiovascular disease while another addressed substance abuse/cannabis use.

#### Country and currency

A majority of the studies were based in the United Kingdom (UK) (9 articles; 27%) with costs expressed in Great British Pounds (GBP). Eight studies were based in European countries including Germany (n = 2), Netherlands (n = 2), Estonia (n = 1), Sweden (n = 1), or a collection of member countries of the European Union (EU) (n = 2) and presented costs in Euros.

Studies from Australia (n = 4), USA (n = 3), Sweden (n = 1), Canada (n = 1), China (n = 1), New Zealand (n = 1), and Singapore (n = 1) conveyed costs in local currency. Six studies converted local currency to United States Dollar (USD), including those from Iran (n = 2), Honduras (n = 1), India (n = 1), Sweden (n = 1) and China (n = 1).

#### Cost estimations and effectiveness outcome measure

A majority of the studies in our review only considered direct costs of the intervention (22 articles; 67%) such as training supplies, materials, or posters. Seven studies (21%) considered both direct and indirect costs. In five studies, the limitations of input costs were unclear or not reported; these studies were still included in the review because they scored above a 80% on the CHEC metric.

The most used effectiveness outcome measures were summary effectiveness units such as quality-adjusted life years (QALYs) (22 articles; 67%) or disability-adjusted life years (DALYs) (4 articles; 12%). Three studies measured health outcomes such as BMI unit reduction (2 articles; 5%), waist circumference, or person years with excess body weight. The remaining studies assessed the avoidance of future health events or conditions such as averted smokers, spared victims of bullying, and averted suicide attempts.

### Technical characteristics

Five technical characteristics extracted from included studies are outlined in detail below. Details for each study included in our review are presented in Additional file 2: Supplementary Material Table 2.

#### Study design

Studies in our review used a variety of modeling approaches. Of the included studies, 15 studies used decision-analytic decision tree models. Markov-based models were used in 10 studies, including six Markov models and four decision analytical Markov models. One study used a microsimulation design, and 11 did not specify the study design used.

#### Time horizon

Most studies used models with designated time horizons of one year (n = 10) or 100 years or lifetime (n = 11). The remaining studies that did report time horizon used years between one to one hundred years (n = 9). Three studies either did not account for time horizon and discounting or simply did not report details regarding the study time horizon; again these studies were included because they obtained a CHEC score of above 80%.

#### Discounting

Selected discount rates ranged from 1.5% to 5% with the most frequent being 3% (12 articles; 36%) followed by 3.5% (6 articles; 18%). Nine studies involved time horizons of one year or less and therefore did not apply discounting. Of the studies, two studies chose not to apply a discount, even though the time horizon of their analysis was two years. And, two studies did not report any detail regarding the use of a discount rate.

#### Sensitivity analysis

Of the included studies, 11 (33%) performed only deterministic sensitivity analyses and 15 (45%) performed both probabilistic sensitivity analyses and deterministic sensitivity analyses. A single study conducted scenario analysis to test sensitivity. We included three studies (9%) that did not perform sensitivity analyses, and it was unclear if sensitivity analyses were conducted for another three studies.

#### Cost-effectiveness threshold

Thresholds determine the level at which an intervention would be considered cost-effective. The standard National Institute for Health and Care Excellence (NICE) UK threshold of £20,000–30,000 was the most common (9 studies). In the US setting, a threshold of $50,000 was utilized in five studies. We documented eight studies that either did not apply a threshold or did not report one in their article.

### Cost-effectiveness of interventions

Almost all school-based interventions were found to be cost-effective (30 articles; 81%). However, six (16%) articles identified interventions that did not demonstrate the cost-effectiveness of interest when compared to the selected comparator. All results in terms of incremental cost-effectiveness ratio (ICER) are presented in Additional file 2: Supplementary Material Table 2. We compare results that analyzed similar interventions and alternatives to summarize the evidence related to cost-effectiveness based on three primary categories; prevention, promotion, and treatment.

#### Prevention

This study documented three studies that assessed prevention strategies to intervene before NCDs occur or identify disease early. These preventive strategies included vaccination and screening and disease identification programs.

*Vaccination*—Offering vaccination against HPV at a school-based setting was found to be a cost-effective intervention in reducing cervical cancer disease across all studies, with the exception of nonavalent vaccines in Singapore [12]. However, the use of nonavalent vaccination was more cost-effective than the use of other vaccinations in Estonia [13]. The authors note that reductions in vaccine prices could further improve the cost-effectiveness of this intervention.

*Screening and disease identification*—The evidence related to screening, identifying, and intervening with students at risk for mental health issues in a school-based setting was mixed. A screening program that subsequently offered focused interventions based on the anxiety level of parents showed improved effectiveness at low costs [14]. The most cost-effective strategy was the one that did not include a screening element, indicating that perhaps the costs of training health professionals to identify the presence of mental health risk factors is not an efficient intervention particularly in the context of a limited timeframe established by the studies.

*Personal awareness*—Increasing awareness of how actions affect health and the development of long-term disease may trigger behavior changes. In six studies, information was shared through programs in schools to increase awareness of a diversity of issues that contribute to NCDs, such as bullying [15], cannabis use [16], tobacco use [17, 18], and nutrition and exercise [19, 20]. In these studies, ICERs were reported on intermediate health outcomes such as victims spared of bullying (14,470 euro), incidental abdominal obesity averted (1515–1993 euro), and smokers avoided ($134). One multicomponent intervention determined an ICER of $1408 per BMI unit avoided [21]. Composite outcome measures showed cost-effectiveness of $275/QALY for nutrition interventions [20]. In another case, the promotion of mental health knowledge and healthy behaviors was more cost-effective than either training professionals at schools or the use of screening tools to identify and intervene with at-risk youth [22].

*Routine physical activity*—Integrating planned routine physical activities, such as dance classes [23], a daily mile of walking [24], or structured physical activity time into the school day were mostly cost-effective; ICERs were well below the established thresholds ranging between $3830/QALY [23] to €19,734/ QALY [25]. The relatively low-cost of implementing such interventions—where minimal training is needed—likely contributed to the cost-effectiveness of this intervention. However, the interventions may be less cost-effective if analyses included the opportunity cost of teaching time. On the other hand, the promotion of walking, cycling or using public transportation to travel to and from school was not a cost-effective obesity prevention measure, as compared to using cars [26].

*Multi-component interventions*—School based-interventions were frequently multi-component in nature, addressing physical activity programming, school policies, and staff training simultaneously. Training school staff in practices to address and manage health risks of students was an important part of these school-based interventions. In our review, six of the studies assessed such programs and documented mixed results. Certain programs showed promising outcomes including Physical Activity 4 Everyone [23], Feel4Diabetes [27] and CHRIPY DRAGON [29]. One particular program, The West Midlands ActiVe lifestyle and healthy Eating in School children (WAVES), targeted both the school and family environment [28, 29]. Even at the low implementation cost, the intervention only demonstrated negligible benefits and sensitivity analyses further demonstrate the high levels of uncertainty related to model inputs. The authors of the study concluded that it is unlikely that such a program would be an efficient use of resources.

#### Treatment

Treatment to manage and care for NCDs were assessed in five studies and included interventions of cognitive behavioral therapy and vitamin D supplements.

*Cognitive behavioral therapy*—In three studies, classroom-based cognitive behavioral therapy (CBT) targeting depression and anxiety symptoms are found to be not cost-effective [30–32]. While the program targeting anxiety reduction found decreased anxiety symptoms at 12 months when delivered by health professionals, it was not maintained at 24 months [12]. In one instance, classroom-CBT delivered by trained facilitators was found to be no more effective at reducing depression symptoms than the usual school provision [33].

*Vitamin D supplements*—We documented two studies published by the same research team indicate that vitamin-D supplementation programs are cost-effective in addressing type 2 diabetes and cardiovascular disease amongst Iranian adolescents with ICERs ranging from $1286- $4803/QALY [34, 35].

## Discussion

This systematic review summarized and described published cost-effectiveness analyses on health promotion interventions for non-communicable chronic diseases, which include elements such as psychoeducation, self-assessment, personal awareness, and active prevention of chronic diseases, in school-based settings. This review identified and included 33 studies that analyze the cost-effectiveness of such interventions.

Our review found evidence to support the value of vaccination, routine physical activity, and supplement delivery interventions. Conversely, evidence cautions against implementing classroom-based CBT (e.g., for mental health) and certain multi-component interventions (e.g., for overweight and obesity). Most interventions in our review required minimal cost inputs such as the implementation of school policies related to nutrition and active living. Such low-cost interventions only require small health gains to be cost-effective.

Heterogeneity of the study characteristics reflects the broad diseases which NCDs encompass. Results of this collection of economic evaluations give a broad conclusion that school-based interventions for chronic disease have favorable cost-effectiveness results. However, the high heterogeneity of the study setting, the targeted disease, and the intervention type means that conclusions should be drawn with caution, and results are not necessarily transferable to different settings.

### Intervention design

Particular characteristics of implementation were analyzed in two studies, including face-to-face versus internet-based delivery of psychological interventions [36] and peer-led versus educator-led smoking education [17]. Results indicated that the manner in which interventions were delivered influenced the efficiency of school-based interventions and should be carefully considered before designing programs. Results broadly indicated that face-to-face delivery by peers may be preferable. Our review also identified studies that engaged with the school-aged children’s wider community, such as parents and families, and yielded success [14, 19, 37–39]. This pattern aligns with findings from the broader literature which indicates that parental involvement is an important determinant for success of school based interventions [40–42]; a recent meta-analysis of school-based anti-bullying program with parental component found a small but statistically significant effect on reducing bullying behavior [43].

### Targeting health conditions in school-based settings

The relatively low cost of many school-based interventions adds to the benefit of leveraging a school-based setting to administer public health promotion interventions. Several health conditions not only have evidence to support cost-effective interventions to ameliorate the condition but also clinical and implementation evidence to suggest the suitability of school-based interventions in improving these health conditions.

*Mental health*. Among the interventions and health conditions included in this review, mental health is an area where significant benefits may be generated through administering the intervention in a school-based setting. First, childhood and adolescence are crucial times for developing foundations for mental health, with onset of many mental health problems occurring in adolescence. As such, primary and secondary school settings provide key environments for delivering healthcare intervention and resources. The benefits of providing mental health intervention in school-setting are numerous, including early identification, accessibility, as well as decreasing disparity in mental health need and service and reducing stigma which are many of the barriers in the way of people seeking mental health treatment [44]. There has been substantial literature and reviews on the efficacy of mental health intervention programs in school-setting. The interventions have targeted a number of different mental health disorders such as depression and anxiety [45–47], developmental disorders [48, 49], bullying [50] and abuse [51], as well as other social/behavioral problems and mediators of poor mental health. Systematic reviews have generally found favorable outcome for school-based intervention, which evidenced small-to-moderate impact of universal interventions on positive mental well-being, mental health disorders, violence and bullying, and pro-social behavior, as well as learning, and behavior and attitudes towards school (i.e., achievement in test scores and school grades, commitment to school, and school attendance) [52]. A meta-analysis on the effectiveness of school-based mental health services in elementary school setting by Sanchez et al. [53] similarly noted small-to-moderate effect size on mental health problems, with “large effects associated with targeted interventions and selective prevention, services that included contingency management, services that were integrated into academic instruction, services that were implemented multiple times per week or daily, and services that targeted externalizing problems”; they emphasized the importance of the roles of school-based personnel on implementation of services [53]. Others have also found targeted intervention to be more effective than universal interventions.

Our systematic review added to these findings and highlighted that these mental health interventions may not only be effective but also cost-effective in various school-based settings. Namely, this review identified that education posters [22], personal awareness [15], psychoeducation [36], dancing [23], screening and intervention [14] are all interventions with strong evidence for cost-effectiveness that may contribute to the mental health of primary and secondary school age individuals. However, it is critical to note the exception that many effective CBT interventions are not cost-effective in school-based settings. Given the inherently structured format of CBT, it can be challenging to efficiently implement it within a school-based environment due to a number of barriers, including needing to fit programs into existing busy schedules, challenges engaging with teachers and students, and cost of training treatment facilitators [30, 32].

*Overweight and Obesity.* Overweight and obesity and related NCDs have become major contributors to global burden of disease in recent years, owing to increasing globalization [54]. Costs associated with the treatment and prevention of obesity and related NCDs can be a burden on healthcare infrastructure and the economy; as such it is especially critical to identify cost-effective strategies for preventing and reducing overweight and obesity.

School-based programs are often considered efficient interventions for obesity prevention as it can also address concerns for undernutrition through the implementation of nutrition-based meal policy, particularly for low-income nations where globalization has led to an increase in overconsumption of cheap and easily accessible, caloric-dense food. Programs often may include components of structured interventions (delivered by professionals or teaching staff), emphasizing lifestyle factors, obesity prevention, education on nutrition, and physical activity. Intervention tends to target modifiable behavioral risk factors for obesity such as caloric consumption, and lack of physical activity, as well as promoting and improving health outcomes by addressing sedentary behavior and diet. However, the effectiveness of school-based intervention programs for obesity, often measured by reduction in BMI, have been variable. Results from meta-analyses of school-based intervention for obesity vary widely. Several analyses concluded that school-based intervention did not appear to be effective in improving BMI [55–58], while other more recent analyses reported mild effectiveness in reducing BMI [59–61].  

This systematic review identified several interventions to reduce the prevalence of overweight and obesity that are cost-effective in a school-based setting. Evidence suggests that multicomponent programs (e.g., APPLE and CATCH), which include elements such as classroom curriculum, a physical education program, and modifications to the school food service are likely cost-effective [37, 62]. More specific nutrition education and personal awareness [20] and health promotion [19] also yield cost-effectiveness in school-based settings.

*Tobacco use.* Approximately 90% of tobacco users begin using before the age of 18 years [63], and it is estimated that half of tobacco users who started using in adolescence continue to use for 15–20 years [64]. The use of tobacco at a young age increases the risk of many diseases among adolescents including respiratory illness, asthma, and reduced pulmonary function [65]. As such, health promotion strategies and school-based tobacco prevention programs designed to reduce smoking behavior in adolescence are thought to be efficacious and cost-effective. The goals of the programs largely focus on education on the risk of tobacco use, social competence interventions (i.e., teaching skills to resist smoking), social influence interventions (i.e., teaching awareness of the social influence that encourages substance use), and multimodal approach taught with parents, teachers, and the community [66]. Our study confirmed that school-based interventions, such as personal awareness training [67] and peer-led intervention [17], can indeed be cost effective in preventing tobacco use.

True cost-effectiveness of interventions are more likely where effects can be sustained into adulthood. Although the studies included in this review used models to estimate lifetime costs and outcomes, none had longitudinal real world data to support assumptions made regarding longer-term impacts of such interventions. It is important for future research to follow students across their lifetime to capture data on the longer term impacts and changes school-based interventions may have in their life course. Furthermore, characterizing heterogeneity and differences in cost-effectiveness that may occur as a result of variations between participants with varying characteristics should be further explored. For example, Breheny et al. [24] found Daily Mile interventions were more cost-effective amongst girls than boys, and it is reasonable to speculate that student income level and other characteristic factors may impact the efficiency of interventions.

### Supplementary Information

**Additional file 1: Table S1.** Search Strategy.

**Additional file 2:** Supplementary Material Table 1 and Supplementary Material Table 2.

## Data Availability

The files used and/or analysed during the current study are available from the corresponding author on reasonable request.

## Abbreviations

BMI: Body mass index
CHEC: Consensus on health economic criteria
CBT: Cognitive behavioral therapy
DALY: Disability-adjusted life year
EU: European Union
GBP: Great British Pounds
HPS: Health promoting school
HPV: Human papillomavirus
ICER: Incremental cost effectiveness ratio
NCD: Non-communicable diseases
NICE: National Institute for Health and Care Excellence
PRISMA: Preferred reporting items for systematic reviews and meta-analyses
QALY: Quality-adjusted life year
UK: United Kingdom
US: United States
USD: United States Dollar
WHO: World Health Organization
