# Supplementary material for: The cost-effectiveness of school-based interventions for chronic diseases: a systematic review
Source: Cost Eff Resour Alloc. 2024 Apr 11;22:26. doi: 10.1186/s12962-024-00511-w (PMC11008027; doi:10.1186/s12962-024-00511-w)
Supplement: Supplementary file 1 — Additional file 1: Table S1. Search Strategy. [file 12962_2024_511_MOESM1_ESM.docx]

**Supplementary 1. Search Strategy**

| *Last modified and run 20 June 2022* | |
| --- | --- |
| **DATABASE** | SEARCH STRINGS |
| **PubMed MEDLINE** | 5 #1 AND #2 AND #3 AND #4  4 ("cost effective analysis"[Text Word] OR "cost effectiveness analysis"[Text Word] OR "cost-effective analysis"[Text Word] OR "cost-effectiveness analysis"[Text Word] OR "cost utility"[Text Word] OR "cost-utility"[Text Word] OR "economic evaluation"[Text Word])  3 (School Health Services OR School Health Promotion [MeSH Terms]) OR (“school*”[Text Word] OR “school-based”[Text Word] OR “schoolchildren”[Text Word] OR “school-aged children”[Text Word] NOT “secondary school*”[Text Word])  2 (Health Promotion OR Self-Assessment OR Health Literacy[MeSH Terms]) OR ("awareness"[Text Word] OR "self-assess"[Text Word] OR "self-assessment"[Text Word] OR "prevent*"[Text Word] OR "health promotion"[Text Word] OR "health intervention"[Text Word] OR "health literacy"[Text Word])  1 (Chronic disease OR Non-communicable diseases OR Mental health [MeSH Terms]) OR ("chronic disease"[Text Word] OR "non-communicable disease*"[Text Word] OR "diabetes"[Text Word] OR "obesity"[Text Word] OR "unbalanced diet"[Text Word] OR "sedentary behaviour"[Text Word] OR "sedentary behavior"[Text Word] OR "dietary factor*"[Text Word] OR "cardiovascular disease"[Text Word] OR "cancer"[Text Word] OR "physical inactivity"[Text Word] OR "chronic disease risk factor"[Text Word] OR "mental health"[Text Word] OR "depression"[Text Word] OR "anxiety"[Text Word]) |
| **Embase** | 1 ‘Chronic disease’/exp OR ‘Non-communicable diseases’/exp OR ‘mental health’/exp  2 (‘chronic disease’ OR 'non-communicable disease?' OR 'diabetes' OR 'smoking' OR 'obesity' OR 'unbalanced diet' OR 'sedentary behaviour' OR 'sedentary behavior' OR 'dietary factor?' OR 'cardiovascular disease' OR 'cancer' OR 'physical inactivity' OR 'chronic disease risk factor' OR ‘mental health’ OR ‘depression’ OR ‘anxiety’)  3 #1 OR #2  4 ‘Health Promotion’/exp OR ‘Self-Assessment’/exp OR ‘Health Literacy’/exp  5 (‘awareness’ OR ‘self-assess’ OR ‘self-assessment’ OR ‘prevent*’ OR ‘health promotion’ OR ‘health intervention’ OR ‘health literacy’)  6 #4 OR #5  7 ‘School Health Services’/exp OR ‘School Health Promotion’/exp  8 ‘school?’ OR ‘school-based’ OR ‘schoolchildren’ OR ‘school-aged children’ OR ‘school setting’ NOT ’secondary school?’  9 #7 OR #8  10 ('cost effective analysis' OR 'cost effectiveness analysis' OR 'cost-effective analysis' OR 'cost-effectiveness analysis' OR 'cost utility analysis' OR 'cost-utility analysis' ‘economic evaluation')  11 #3 AND #6 AND #9 AND #10  12 #11 NOT ‘conference abstract’/it  13 #12 NOT ‘letter’/it  14 #13 NOT ‘editorial’/it  15 #14 NOT ‘conference review’/it  16 #15 NOT ‘erratum’/it  17 #16 NOT ‘note’/it |
| **Web of Science** | 6 #5 NOT DT=(Note OR Letter OR Meeting Abstract OR News Item OR Editorial Material)  5 #1 AND #2 AND #3 AND #4  4 ALL = ("cost effective analysis" OR "cost effectiveness analysis" OR "cost-effective analysis" OR “cost-effectiveness analysis" OR "economic evaluation" OR "economic evaluations" OR "cost utility" OR "cost-utility”)  3 ALL = (“school health services” OR “school” OR “school-based” OR “schoolchildren” OR “school-aged children” OR “school setting” NOT “secondary school”)  2 ALL = (“health promotion” OR “self-assessment” OR “awareness” OR “self-assess” OR “prevention” OR “health intervention” OR “health literacy” )  1 ALL = ( “chronic disease” OR “non-communicable disease” OR “diabetes” OR “obesity” OR “unbalanced diet” OR “sedentary behaviour” OR “sedentary behavior” OR “dietary factor” OR “cardiovascular disease” OR “cancer” OR “physical inactivity” OR “chronic disease risk factor” OR “depression” OR “anxiety” ) |
| **Cochrane** | #1 Chronic disease OR Non-communicable diseases OR Mental Health [MeSH] explode all trees  #2 (“chronic disease” OR “non-communicable disease*” OR “diabetes” OR “smoking” OR “obesity” OR “unbalanced diet” OR “sedentary behaviour” OR “sedentary behavior” OR “dietary factor*” OR “cardiovascular disease” OR “cancer” OR “physical inactivity” OR “chronic disease risk factor” OR “mental health” OR “depression” OR “anxiety” ): ti,ab,kw  #3 #1 OR #2  #4 Health Promotion OR Self-Assessment OR Health Literacy [MeSH] explode all trees  #5 ( “awareness” OR “self-assess” OR “self-assessment” OR “prevent*” OR “health promotion” OR “health intervention” OR “health literacy”): ti,ab,kw  #6 #4 OR #5  #7 School Health Services OR School Health Promotion [MeSH] explode all trees  #8 (“school*” OR “school-based” OR “schoolchildren” OR “school-aged children” OR “school setting” NOT “secondary school”)  #9 #7 OR #8  #10 Cost-Benefit Analysis [MeSH] explode all trees  #11 (“cost effective” OR “cost effectiveness” OR “economic evaluation” OR “economic evaluations” OR “cost utility” ): ti,ab,kw  #12 #10 OR #11  #13 #3 AND #6 AND #9 AND #12 |
