# Supplementary material for: The cost-effectiveness of school-based interventions for chronic diseases: a systematic review
Source: Cost Eff Resour Alloc. 2024 Apr 11;22:26. doi: 10.1186/s12962-024-00511-w (PMC11008027; doi:10.1186/s12962-024-00511-w)
Supplement: Supplementary file 2 — Additional file 2: Supplementary Material Table 1 and Supplementary Material Table 2. [file 12962_2024_511_MOESM2_ESM.docx]

**Supplementary Material 2**

**Table 1. Quality Assessment**

| Author (Year) | Is the study population clearly described? | Are competing alternatives clearly described? | Is a well-defined research question posed in answerable form? | Is the economic study design appropriate to the stated  objective? | Is the chosen time horizon appropriate in order to include  relevant costs and consequences? | Is the actual perspective chosen appropriate? | Are all important and relevant costs for each alternative  identified? | Are all costs measured appropriately in physical units? | Are costs valued appropriately? | Are all important and relevant outcomes for each alternative  identified? | Are all outcomes measured appropriately? | Are outcomes valued appropriately? | Is an incremental analysis of costs and outcomes of alternatives  performed? | Are all future costs and outcomes discounted appropriately? | Are all important variables, whose values are uncertain,  appropriately subjected to sensitivity analysis? | Do the conclusions follow from the data reported? | Does the study discuss the generalizability of the results to  other settings and patient/client groups? | Does the article indicate that there is no potential conflict of  interest of study researcher(s) and funder(s)? | Are ethical and distributional issues discussed appropriately? | TOTAL SCORE (OUT OF 19) | % achieved |
| --- | --- | --- | --- | --- | --- | --- | --- | --- | --- | --- | --- | --- | --- | --- | --- | --- | --- | --- | --- | --- | --- |
| Adab 2018 | Y | Y | Y | Y | Y | Y | Y | Y | Y | Y | Y | Y | Y | Y | Y | Y | Y | N | N | 17 | 89 |
| Aguilar 2015 | Y | Y | Y | Y | Y | Y | N | Y | Y | Y | Y | Y | Y | Y | N | Y | N | Y | Y | 16 | 84 |
| Ahern 2018 | Y | Y | Y | Y | Y | Y | Y | Y | Y | Y | Y | Y | Y | Y | Y | Y | Y | Y | Y | 19 | 100 |
| Anderson 2014 | Y | Y | Y | Y | Y | Y | Y | Y | Y | Y | Y | Y | Y | Y | N | Y | Y | Y | Y | 18 | 95 |
| Beckman 2015 | Y | Y | Y | Y | Y | Y | Y | Y | Y | Y | Y | Y | Y | Y | Y | Y | Y | N | N/A | 17 | 89 |
| Blakely 2014 | Y | Y | Y | Y | Y | Y | Y | Y | Y | Y | Y | Y | Y | Y | Y | Y | Y | Y | Y | 19 | 100 |
| Breheny 2020 | Y | Y | Y | Y | N | Y | Y | Y | Y | Y | Y | Y | Y | N | Y | Y | N | Y | Y | 16 | 84 |
| Brown3rd 2013 | Y | N | Y | Y | Y | N | N | Y | Y | Y | Y | Y | Y | N | Y | Y | Y | N | Y | 14 | 74 |
| BrownIII 2007 | Y | Y | Y | Y | Y | Y | Y | Y | Y | Y | Y | Y | Y | Y | Y | Y | N | N | Y | 17 | 89 |
| Canaway 2019 | Y | Y | Y | Y | Y | Y | Y | Y | Y | Y | Y | Y | Y | Y | N | Y | N | Y | Y | 17 | 89 |
| Conesa 2018 | Y | Y | Y | Y | Unclear | Y | Y | Y | Y | Y | Unclear | Unclear | Y | N | Y | Y | Y | Y | Y | 15 | 79 |
| Conner 2019 | Y | Y | Y | N | Y | N | Y | Y | Y | Y | Y | Y | Y | Y | N | Y | N | N | Y | 14 | 74 |
| Deogan 2015 | Y | N | Y | Y | Y | Y | Y | Y | Y | Y | Y | Y | Y | Y | Y | Y | Y | Y | N | 17 | 89 |
| Dino 2008 | Y | Y | Y | Y | Y | Y | N | Y | Y | Y | Y | Y | Y | N | Y | Y | Y | N | N | 15 | 79 |
| Ekwaru 2017 | Y | Y | Y | Y | Y | Y | N | Y | Y | Y | Y | Y | Y | Y | Y | Y | Y | Y | Y | 18 | 95 |
| Ekwaru 2020 | Y | Y | Y | Y | Y | N | Unclear | Y | Y | Y | Y | Y | Y | N | Y | Y | N | Y | N | 14 | 74 |
| Ekwaru 2021 | Y | N | Y | Y | Y | Y | Unclear | Y | Y | Y | Y | Y | Y | N | Y | Y | N | Y | Y | 15 | 79 |
| Ford 2019 | Y | Y | Y | Y | Y | Y | Y | Y | Y | Y | Y | Y | Y | Y | Y | Y | N | Y | Y | 18 | 95 |
| Garmy 2019 | Y | Y | Y | N | N | N | N | N | N | N | Y | Y | Y | N | N | Y | Y | N | Y | 9 | 47 |
| Graziose 2017 | Y | Y | Y | Y | Y | Y | Y | Y | Y | Y | Y | Y | Y | Y | Y | Y | Y | Y | N | 18 | 95 |
| Hollingworth 2012 | Y | Y | Y | Y | Y | Y | Y | Y | Y | Y | Y | Y | Y | Y | Y | Y | Y | Y | Y | 19 | 100 |
| Jadambaa 2022 | Y | Y | Y | Y | Y | Y | Y | Y | Y | Y | Y | Y | Y | Y | Y | Y | Y | Y | N | 18 | 95 |
| Kesztyüs 2013 | Y | Y | Y | Y | Y | Y | Y | Y | N | Y | Y | Y | Y | Y | Y | Y | Y | N | Y | 17 | 89 |
| Kesztyüs 2017 | Y | Y | Y | Y | Y | Y | Y | Y | Y | Y | Y | Y | Y | Y | Y | Y | Y | N | Y | 18 | 95 |
| Lee 2017 | Y | Y | Y | Y | Y | Y | Y | Y | Y | Y | Y | Y | Y | Y | Y | Y | Y | Y | Y | 19 | 100 |
| Legood 2021 | Y | Y | Y | Y | Y | Y | Y | Y | Y | Y | Y | Y | Y | Y | Y | Y | Y | Y | N | 18 | 95 |
| Li 2019 | Y | Y | Y | Y | N | Y | N | N | N | Y | Y | Y | Y | N | N | Y | Y | N | Y | 12 | 63 |
| Meng 2013 | Y | Y | Y | Y | N | Y | Y | Y | Y | Y | Y | Y | Y | N | N | Y | Y | N | Y | 15 | 79 |
| Mihalopoulos 2012 | Y | Y | Y | N | Y | Y | N | N | N | Y | Y | Y | Y | Y | Y | Y | Y | N | N | 13 | 68 |
| Moodie 2011 | Y | Y | Y | Y | Y | Y | Y | Y | Y | Y | Y | Y | Y | Y | Y | Y | Y | N | N | 17 | 89 |
| Oosterhoff 2020 | Y | Y | Y | Y | Y | Y | Y | Y | Y | Y | Y | Y | Y | Y | Y | Y | Y | Y | Y | 19 | 100 |
| Philipsson 2013 | Y | Y | Y | Y | Y | Y | Y | Y | Y | Y | Y | Y | Y | Y | Y | Y | Y | N | Y | 18 | 95 |
| Phua 2021 | Y | Y | Y | Y | Y | Y | Y | Y | Y | Y | Y | Y | Y | Y | Y | Y | Y | Y | Y | 19 | 100 |
| Ross 2006 | Y | Y | Y | Y | N | N | N | Y | Y | Y | Y | Y | Y | Y | Y | Y | Y | Y | N | 15 | 79 |
| Rush 2014 | Y | N | Y | N | Y | Y | N | Y | Y | N | Y | Y | Y | Y | Y | Y | N | N | N | 12 | 63 |
| Simon 2013 | Y | Y | Y | Y | N | Y | Y | Y | Y | Y | Y | Y | Y | Y | Y | Y | Y | Y | N | 17 | 89 |
| Stallard 2013 | Y | Y | Y | Y | Y | Y | Y | Y | Y | Y | Y | Y | Y | Y | Y | Y | Y | N | Y | 18 | 95 |
| Stallard 2015 | Y | Y | Y | Y | Y | Y | Y | Y | Y | Y | Y | Y | Y | Y | Y | Y | N | N | Y | 17 | 89 |
| Sutherland 2016 | Y | Y | Y | Y | Y | Y | Y | Y | Y | Y | Y | Y | Y | N | Y | Y | N | Y | Y | 17 | 89 |
| Tengs 2001 | Y | N | Y | Y | Y | Y | Y | Y | Y | N | Y | Y | Y | Y | Y | Y | N | N | N | 14 | 74 |
| Vijgen 2008 | N | N | Y | Y | Y | Y | N | Y | Y | N | Y | Y | N | Y | Y | Y | N | Y | N | 12 | 63 |
| Võrno 2017 | Y | Y | Y | Y | Y | Y | Y | Y | Y | Y | Y | Y | Y | Y | Y | Y | N | N | N | 16 | 84 |
| Wang 2001 | Y | N | Y | Y | Y | Y | N | Y | Y | N | Y | Y | N | Y | Y | Y | N | N | N | 12 | 63 |
| Wang 2003 | Y | N | Y | Y | Y | Y | N | Y | Y | N | Y | Y | N | Y | Y | Y | Y | N | N | 13 | 68 |
| Wang 2008 | Y | Y | Y | Y | Y | Y | Y | Y | Y | Y | Y | N | N | N | Y | Y | N | N | N | 13 | 68 |
| Willems 2021 | Y | Y | Y | Y | Y | Y | N | Y | Y | Y | Y | Y | Y | Y | Y | Y | N | Y | Y | 17 | 89 |
| Xu 2020 | Y | Y | Y | Y | Y | Y | Y | Y | Y | Y | Y | Y | Y | Y | Y | Y | N | Y | Y | 18 | 95 |
| Zandieh 2021 | Y | Y | Y | Y | Y | Y | Y | Y | Y | Y | Y | Y | Y | N | Y | Y | Y | Y | N | 17 | 89 |
| Zandieh 2022 | Y | Y | Y | Y | Y | Y | Y | Y | Y | Y | Y | Y | Y | N | Y | Y | N | Y | Y | 17 | 89 |
| Zanganeh 2021 | Y | N | Y | Y | Y | Y | N | Y | Y | Y | Y | Y | Y | Y | Y | Y | Y | N | Y | 16 | 84 |

**Table 2. Study Characteristics**

| **Study** | **Study Characteristics** | | **Results** |
| --- | --- | --- | --- |
|  | ***Descriptive characteristics*** | ***Technical characteristics*** |  |
| Adab (2018) | Perspective: Public Sector  Intervention/Comparator: Multi component intervention WAVES / No intervention  Setting: Primary school  Targeted Disease: Obesity/Overweight  Country/Currency (adj. year): UK;GBP (2014)  Cost estimates: Direct  Outcome measure: QALY | Modeling approach: Not reported  Time horizon: 30 months  Discounting: 3.5%  Sensitivity analysis: Deterministic  Threshold used: £20,000–30,000/QALY | Results: £46,083/QALY  Author's conclusions: With respect to the analysis of effectiveness and cost-effectiveness, the main result is the lack of intervention effect in terms of QALY gain or in terms of obesity prevention. The economic evaluation suggests that the intervention, despite its relatively low cost on a per-child basis, has negligible benefits ,and thus fails to demonstrate cost-effectiveness.  % EE assessment satisfied: 89% |
| Aguilar (2015) | Perspective: Societal and Government  Intervention/Comparator: Vaccination / No intervention  Setting: School +  Targeted Disease: HPV  Country/Currency (adj. year): Honduras;USD (2013)  Cost estimates: Direct; Non-direct  Outcome measure: DALY | Modeling approach: Markov decision analytic model  Time horizon: Lifetime  Discounting: 3%  Sensitivity analysis: Other: Scenario analysis  Threshold used: 2339 | Results: $926/DALY (government) $843/DALY (societal)  Author's conclusions: When comparing the costs and benefits of HPV vaccine introduction in Honduras, it is clear that this intervention would be highly cost-effective and that the intervention would greatly reduce cervical cancer disease.  % EE assessment satisfied: 84% |
| Ahern (2018) | Perspective: Payor  Intervention/Comparator: Screening + intervention / Educational posters  Setting: N/A  Targeted Disease: Mental health (Suicide)  Country/Currency (adj. year): Other: EU countries (Austria, Estonia, France, Germany, Hungary, Ireland, Italy ,Romania, Slovenia and Spain);EUR (2010)  Cost estimates: Direct  Outcome measure: Suicide attempt and severe suicidal ideation with suicide plans. | Modeling approach: Not reported  Time horizon: 1 yr  Discounting: None  Sensitivity analysis: Deterministic; Probabilistic  Threshold used: | Results: Suicide attempt  QPR: €120,567/QALY  YAM: €47,017/QALY  ProfScreen: €64,050/ QALY  Severe suicide ideation  QPR: dominated  YAM: €48,216/QALY  ProfScreen: €108,790/QALY  Author's conclusions: This CEA supports YAM as the most cost-effective of the SEYLE interventions in preventing both a suicide attempt and severe suicidal ideation  % EE assessment satisfied: 100% |
| Anderson (2014) | Perspective: Healthcare  Intervention/Comparator: Psychoeducation (Cognitive behavioural therapy) / Usual education  Setting: Secondary school  Targeted Disease: Mental health  Country/Currency (adj. year): UK;GBP (2010)  Cost estimates: Direct  Outcome measure: QALY | Modeling approach: Not reported  Time horizon: 1 yr  Discounting: None  Sensitivity analysis: None  Threshold used: £20,000/QALY | Results: CBT is more costly and less effective than usual school provision  Author's conclusions: Our analysis suggests that the universal provision of classroom-based CBT is unlikely to be either more effective or less costly than usual school provision.  % EE assessment satisfied: 95% |
| Beckman and Svensson (2015) | Perspective: Public Sector  Intervention/Comparator: Personal awareness/ Health promotion / No intervention  Setting: Secondary school  Targeted Disease: Mental health (bullying)  Country/Currency (adj. year): Sweden;SEK (2014)  Cost estimates: Direct  Outcome measure: Spared victim of bullying | Modeling approach: Decision analytic model  Time horizon: 3 yrs  Discounting: 3%  Sensitivity analysis: Deterministic; Probabilistic  Threshold used: 585 000 SEK (Euro 64 500). | Results: 131 250 Swedish kronor (euro 14 470) per victim spared  Author's conclusions: Using a relevant willingness-to-pay threshold shows that the OBPP is a cost-effective intervention  % EE assessment satisfied: 89% |
| Blakely (2014) | Perspective: Healthcare  Intervention/Comparator: Vaccination / No intervention  Setting: N/A  Targeted Disease: HPV  Country/Currency (adj. year): New Zealand;New Zealand dollars (2011)  Cost estimates: Direct  Outcome measure: QALY | Modeling approach: Markov model  Time horizon: Lifetime  Discounting: 3%  Sensitivity analysis: Deterministic  Threshold used: NZ$40,000/QALY | Results: NZ$18,800/QALY  Author's conclusions: Reductions in vaccine price will greatly improve cost-effectiveness of all options, possibly making a law for mandatory vaccination optimal from a health sector perspective.  % EE assessment satisfied: 100% |
| Breheny (2020) | Perspective: Public Sector  Intervention/Comparator: Structured physical activity intervention / No intervention  Setting: Primary school  Targeted Disease: Obesity/Overweight  Country/Currency (adj. year): UK;GBP (2017)  Cost estimates: Direct  Outcome measure: QALY | Modeling approach: Not reported  Time horizon: 1 yr  Discounting: Not reported  Sensitivity analysis: Probabilistic  Threshold used: £20,000/QALY | Results: £7,455.21/QALY or £2,492/QALY (girls)  Author's conclusions: Overall the Daily Mile had a small but non-significant effect on BMIz, however, it had a greater effect in girls suggesting that it might be considered as a cost-effective component of a system-wide approach to childhood obesity prevention.  % EE assessment satisfied: 84% |
| Brown (2013) | Perspective: Not reported  Intervention/Comparator: Personal awareness/ Health promotion / No intervention  Setting: Secondary school  Targeted Disease: Tobacco use  Country/Currency (adj. year): India;USD (2006)  Cost estimates: Direct  Outcome measure: QALY | Modeling approach: Markov decision analytic model  Time horizon: 12 years  Discounting: Not reported  Sensitivity analysis: Probabilistic  Threshold used: $50,000/QALY | Results: $2057/QALY  Author's conclusions: Our results show that MYTRI is a cost-effective program, costing just $2769 perquality-adjusted life-year.  % EE assessment satisfied: 89% |
| Brown (2007) | Perspective: Societal  Intervention/Comparator: / Control groups  Setting: N/A  Targeted Disease: Obesity/Overweight  Country/Currency (adj. year): United States;USD (2004)  Cost estimates: Direct; Non-direct  Outcome measure: QALY | Modeling approach: Decision analytic model  Time horizon: Lifetime  Discounting: 3%  Sensitivity analysis: Probabilistic  Threshold used: $30,000/QALY | Results: $900/QALY  Author's conclusions: Childhood school-based programs such as CATCH are beneficial investments  % EE assessment satisfied: 89% |
| Canaway (2019) | Perspective: Public Sector  Intervention/Comparator: Multi component intervention WAVES / Usual activities  Setting: Primary school  Targeted Disease: Obesity/Overweight  Country/Currency (adj. year): UK;GBP (2014)  Cost estimates: Direct  Outcome measure: QALY | Modeling approach: Not reported  Time horizon: 30 months  Discounting: 3.5%  Sensitivity analysis: Deterministic  Threshold used: £30,000/QALY | Results: £26,815/QALY  Author's conclusions: At first glance the results of this economic evaluation appear to show the WAVES intervention to be a cost-effective use of public resources, however there are high levels of uncertainty evidenced by the low probability of cost effectiveness at varying threshold levels of willingness to pay.  % EE assessment satisfied: 89% |
| Deogan (2015) | Perspective: Societal  Intervention/Comparator: Personal awareness / Usual education  Setting: N/A  Targeted Disease: Cannabis smoking  Country/Currency (adj. year): Sweden;EUR (2014)  Cost estimates: Direct; Non-direct  Outcome measure: QALY | Modeling approach: Markov model  Time horizon: Lifetime  Discounting: 3%  Sensitivity analysis: Deterministic  Threshold used: €50,000/QALY | Results: €22,384/QALY  Author's conclusions: School-based prevention such as Project ALERT has the potential to be cost-effective and to be cost-saving if implemented in deprived areas.  % EE assessment satisfied: 95% |
| Ekwaru, John Paul (2017) | Perspective: School system  Intervention/Comparator: Multi component intervention / No intervention  Setting: Primary school  Targeted Disease: 3 weight status categories and 13 chronic diseases with established links to weight status  Country/Currency (adj. year): Canada;Canadian dollar 2008  Cost estimates: Direct  Outcome measure: person years of excess body weight, person years of obesity, person years with chronic diseases, and QALY | Modeling approach: Markov model  Time horizon: future costs 10 years and health outcomes (up to 84 years)  Discounting: 3%  Sensitivity analysis: Probabilistic  Threshold used: CA$50,000/QALY and CA$100,000/QALY | Results: CA$33,421 per QALY gained, and CA$1,555, CA$1,709 and CA$14,218 per prevented person years of excess weight, obesity and chronic disease, respectively.  Author's conclusions: These estimates show that APPLE Schools is cost effective at a threshold of ICER<CA$50,000  % EE assessment satisfied: 95% |
| Ford (2019) | Perspective: Public Sector  Intervention/Comparator: Training teachers / Usual eduation  Setting: School +  Targeted Disease: Strengths and Difficulties Questionnaire Total Difficulties score  Country/Currency (adj. year): UK;GBP (2014)  Cost estimates: Direct; Non-direct  Outcome measure: SDQ-TD score, QALYs | Modeling approach: Decision analytic model  Time horizon:  Discounting: 3.5%  Sensitivity analysis:  Threshold used: £20,000/QALY | Results: 16000/QALY  Author's conclusions: Although the results of the clinical analysis alone do not support the hypothesis that TCM is more effective than TAU, the primary cost-effectiveness analysis, comparing outcomes in terms of total SDQ-TD scores, suggested that TCM may be cost-effective compared with TAU at 30 months for a relatively wide range of values of willingness to pay for improvements in SDQ-TD score.  % EE assessment satisfied: 95% |
| Graziose (2017) | Perspective: Societal  Intervention/Comparator: Personal awareness/ Health promotion / No intervention  Setting: Primary school  Targeted Disease: Obesity/Overweight  Country/Currency (adj. year): United States;USD (2012)  Cost estimates: Direct  Outcome measure: QALY | Modeling approach: Decision analytic model  Time horizon: Lifetime  Discounting: 3%  Sensitivity analysis: Deterministic  Threshold used: $50,000/QALY | Results: $275/QALY (95% confidence interval, –$2,576/QALY to$2,084/QALY) with estimates up to $6,029/QALY in sensitivity analyses  Author's conclusions: This cost-effectiveness model suggests that a nutrition education curriculum in public schools is effective and cost-effective in reducing childhood obesity, consistent with the authors’ hypothesis and previous literature. Future research should assess the feasibility and sustainability of scale-up  % EE assessment satisfied: 100% |
| Hollingworth (2011) | Perspective: Public Sector  Intervention/Comparator: Peer-led intervention / Usual care  Setting: Secondary school  Targeted Disease: Tobacco use  Country/Currency (adj. year): UK;GBP(2008)  Cost estimates: Direct  Outcome measure: odds ratio for being a smoker | Modeling approach: Not reported  Time horizon: 1 yr  Discounting: no discounting as intervention occurred during one school year  Sensitivity analysis: Deterministic  Threshold used: n/a | Results: The incremental cost per student not smoking at 2 years was £1,500 (95% CI = £669–£9,947).  Author's conclusions: A peer-led intervention reduced smoking among adolescents at a modest cost. The intervention is cost-effective under realistic assumptions regarding the extent to which reductions in adolescent smoking lead to lower smoking preva-lence and/or earlier smoking cessation in adulthood.  % EE assessment satisfied: 95% |
| Jadambaa (2022) | Perspective: Societal  Intervention/Comparator: Personal awareness / Parent involvement / Usual activities  Setting: Primary school  Targeted Disease: DALY, intermediate events modelled included anxiety disorders, depressive disorders, intentional self-harm, cost-savings accrued by educator time, and reduced productivity losses for carers associated with absenteeism. Uncertainty analysis and scenario analyses were also conducted  Country/Currency (adj. year): Australia;AUD (2016)  Cost estimates: Direct  Outcome measure: DALY | Modeling approach: Decision analytic model  Time horizon: 10 yrs  Discounting: 3.5% applied to costs  Sensitivity analysis: Probabilistic  Threshold used: $50,000/DALY | Results: A$ 1646/ DALY averted  Author's conclusions: The Friendly Schools Friendly Families anti-bullying intervention represents a good investment compared to usual activities for the management of child and adolescent bullying in Australia.  % EE assessment satisfied: 89% |
| Kesztyüs (2011) | Perspective: Societal  Intervention/Comparator: Health promotion / No intervention  Setting: Primary school  Targeted Disease: Obesity/Overweight  Country/Currency (adj. year): Germany;EUR (2008)  Cost estimates: Direct  Outcome measure: Waist circumference | Modeling approach: Not reported  Time horizon: 1 yr  Discounting: no discounting were applied due to short intervention period of one year  Sensitivity analysis: Deterministic  Threshold used: n/a | Results: ICER was €11.11(95% confidence interval (CI) [8.78; 15.02]) per cm WC and€18.55 (95% CI [14.04; 26.86]) per unit WHtR gain prevented.  Author's conclusions: Assuming maximum willingness to pay of €35 the intervention is cost-effective with a positive net monetary benefit.  % EE assessment satisfied: 95% |
| Kesztyüs (2017) | Perspective: Societal  Intervention/Comparator: Personal awareness/ Health promotion / N/A  Setting: Primary school  Targeted Disease: abdominal obesity  Country/Currency (adj. year): Germany;EUR(2010)  Cost estimates: Direct  Outcome measure: incidence of abdominal obseity averted | Modeling approach: Not reported  Time horizon: 1 yr  Discounting: no discounting as the time horizon is one year  Sensitivity analysis: Deterministic  Threshold used: | Results: The cost per incidental abdominal obesity averted varied between €1515 and €1993, depending on the size of the observed target group  Author's conclusions: This study demonstrates the positive effects of state-wide, school-based health promotion on incidental abdominal obesity, at affordable costs and with proven cost-effectiveness.  % EE assessment satisfied: 100% |
| Lee (2017) | Perspective: Public Sector  Intervention/Comparator: Psychoeducation / Face to face vs internet  Setting: Secondary school  Targeted Disease: Mental health (i.e., depression)  Country/Currency (adj. year): Australia;AUD (2013)  Cost estimates: Direct  Outcome measure: DALY | Modeling approach: Markov decision analytic model  Time horizon: 10 yrs  Discounting: 3%  Sensitivity analysis: Probabilistic  Threshold used: $50,000/ DALY | Results: $7350 per DALY averted (95% uncertainty interval (UI): dominates–23 070) for universal prevention, and $19 550 per DALY averted (95% UI: 3081–56 713) for indicated prevention.  Author's conclusions: School-based psychological interventions appear to be cost-effective. However, realising efficiency gains in the population is ultimately dependent on ensuring successful system-level implementation.  % EE assessment satisfied: 95% |
| Legood (2021) | Perspective: Public Sector  Intervention/Comparator: Training teachers / No intervention  Setting: Secondary school  Targeted Disease: CHU-9D measure, quality of life  Country/Currency (adj. year): UK;GBP  Cost estimates: Direct  Outcome measure: QALY | Modeling approach: Decision analytic model  Time horizon: 3 yrs  Discounting: 3.5%  Sensitivity analysis: Deterministic  Threshold used: The NICE threshold of £20,000 to £30,000 per QALY | Results: Year 2: £13,284/QALY. Year 3: £1,875/QALY  Author's conclusions: This study provides strong evidence collected prospectively from a randomized study that this school-based intervention is highly cost-effective. Education- and health-sector policy makers should consider investment in scaling up this intervention  % EE assessment satisfied: 89% |
| Moodie (2011) | Perspective: Societal  Intervention/Comparator: Physical activity--travel / N/A  Setting: N/A  Targeted Disease: Obesity/Overweight  Country/Currency (adj. year): Australia;AUD (2001)  Cost estimates: Direct  Outcome measure: DALY | Modeling approach: Not reported  Time horizon: rest of life or 100 years  Discounting: 3%  Sensitivity analysis: Deterministic  Threshold used: $AUD50,000 per DALY | Results: $AUD117,000 per DALY saved  Author's conclusions: The intervention was not cost-effective as an obesity prevention measure under base-run modeling assumptions. The attribution of some costs to non-obesity objectives would be justified given the program’s multiple benefits. Cost-effectiveness would be further improved by considering the wider school community impacts  % EE assessment satisfied: 100% |
| Oosterhoff (2020) | Perspective: Societal  Intervention/Comparator: Structured physical activity intervention / Usual education  Setting: N/A  Targeted Disease: chronic diseases  Country/Currency (adj. year): Netherlands;EUR (2018)  Cost estimates: Direct  Outcome measure: QALY | Modeling approach: Not reported  Time horizon: Lifetime  Discounting: 1.5%, 4  Sensitivity analysis: Probabilistic  Threshold used: €20,000/QALY | Results: €19,734/ QALY gained  Author's conclusions: HPSF may be a cost-effective and equitable strategy for combatting the lifetime burden of unhealthy lifestyles. The win-win situation will, however, only be realised if the intervention effect is sustained into adulthoodf or all SES groups  % EE assessment satisfied: 95% |
| Philipsson (2013) | Perspective: Societal  Intervention/Comparator: Physical activity (dance) / No intervention  Setting: Secondary school  Targeted Disease: Mental health (i.e., internalizing problems)  Country/Currency (adj. year): Sweden;USD (2011)  Cost estimates: Other: Unclear  Outcome measure: QALY | Modeling approach: Decision analytic model  Time horizon: 20 months  Discounting: 3%  Sensitivity analysis: Other: Unclear  Threshold used: 75000 | Results: $3,830 per QALY  Author's conclusions: For adolescent girls with internalizing problems, it can be cost-effective to complement the school health services with a dance intervention which was shown to increase QOL with low costs per QALY (assume the effect remains over time).  % EE assessment satisfied: 100% |
| Phua (2021) | Perspective: Healthcare  Intervention/Comparator: Vaccination / Nonavalent vs bivalent  Setting: Secondary school  Targeted Disease: HPV  Country/Currency (adj. year): Singapore;Singapore dollars (S$)  Cost estimates: Direct  Outcome measure: QALY | Modeling approach: Markov Model  Time horizon: 100 years  Discounting: 3%  Sensitivity analysis: Probabilistic  Threshold used: None | Results: S$61,629/QALY  Author's conclusions: From a healthcare system’s perspective, the nonavalent vaccine was not a cost-effective option compared with the bivalent vaccine at their current prices when used as part of the national school-based HPV vaccination program for 13-year old female students in Singapore, especially when the bivalent vaccine was shown to be cost-saving compared with no vaccination program and the base case ICER of S$61,629 exceeded ICERs of drugs which previously received positive subsidy recommendations for the treatment of chronic diseases in Singapore (ranging from dominance to <S$45,000/QALY).  % EE assessment satisfied: 89% |
| Simon (2013) | Perspective: Societal  Intervention/Comparator: Screening + intervention / No intervention  Setting: Primary school  Targeted Disease: Mental health (i.e., anxiety)  Country/Currency (adj. year): Netherlands;EUR (2012)  Cost estimates: Direct; Non-direct; Other: out-of pocket costs (e.g. over-the-counter medication)  Outcome measure: QALY non-interpretable | Modeling approach: Decision analytic model  Time horizon: 2 years  Discounting: 4%  Sensitivity analysis: Deterministic  Threshold used: €3,000 extra per improved child | Results: The expected effects were 0.19 for strategies 1 and 3, and 0.18 for strategies 2 and 4. The expected costs were lowest for strategy 4 (€1,295.99) and highest for strategy 1 (€1,311.10).Due to higher costs and lower effectiveness, strategies 1 and 2were dominated by strategies 3 and 4, respectively. This means that strategies 1 and 2 are not cost-effective and should be discarded. The incremental (extra) costs of strategy 3 compared to strategy 4 were€107 per ‘‘ADIS improved’’ child, which means that implementation of strategy 3,‘‘screening and offering child- or parent-focused prevention based on parental anxiety’’, compared to strategy 4, ‘‘do nothing’’, requires a societal investment of€100 for each additional ‘‘ADIS improved’’ child.  Author's conclusions: Screening, followed by offering the parent-focused intervention to children of anxious parents, and the child-focused intervention to children of non-anxious parents had the highest expected incremental effects at low incremental costs compared to ‘‘do nothing’’ (i.e. no screening and no preventive intervention offered).  % EE assessment satisfied: 95% |
| Stallard (2013) | Perspective: Societal  Intervention/Comparator: Psychoeducation (Cognitive behavioural therapy) / Usual education  Setting: Secondary school  Targeted Disease: Mental health (i.e., depression)  Country/Currency (adj. year): UK;GBP  Cost estimates: Other: Unclear  Outcome measure: QALY | Modeling approach: Other: Regression model  Time horizon: 1 yr  Discounting: None  Sensitivity analysis: Other: Unclear  Threshold used: £20,000 per QALY | Results: Given the small and highly uncertain differences in cost and effectiveness (SMFQ or QALYs) between classroom-based CBT and attention control PHSE, it would not be informative to estimate the incremental cost-effectiveness of these comparisons.  Author's conclusions: Classroom-based CBT, attention control PSHE and usual PSHE produced similar outcomes.  % EE assessment satisfied: 89% |
| Stallard (2015) | Perspective: Healthcare  Intervention/Comparator: Psychoeducation (Cognitive behavioural therapy) / Usual care  Setting: Primary school  Targeted Disease: Mental health (i.e., anxiety)  Country/Currency (adj. year): UK;GBP  Cost estimates: Other: Unclear  Outcome measure: QALY | Modeling approach: Decision analytic model  Time horizon: 2 years  Discounting: None  Sensitivity analysis: Other: Unclear  Threshold used: £30,000 / QALY | Results: £5600 per QALY at 12 months  Author's conclusions: Although greater reductions in anxiety were noted at 12 months when the FRIENDS programme was delivered by health leaders, these additional benefits were not maintained at 24 months.  % EE assessment satisfied: 89% |
| Sutherland (2016) | Perspective: Societal  Intervention/Comparator: Multi component intervention / No intervention  Setting: Secondary school  Targeted Disease: Physical health  Country/Currency (adj. year): Australia;AUD  Cost estimates: Direct  Outcome measure: BMI unit avoided | Modeling approach: Decision analytic model  Time horizon: 2 years  Discounting: N/A  Sensitivity analysis: None  Threshold used: None used | Results: $1,408 ($788–$6,570) per BMI unit avoided  Author's conclusions: The PA4E1 intervention had a statistically significant intervention effect on physical activity and weight gain which can be achieved for a relatively low monetary cost of $394AUD per student over a 24-month period.  % EE assessment satisfied: 84% |
| Võrno (2017) | Perspective: Healthcare  Intervention/Comparator: Vaccination / Nonavalent vs bivalent  Setting: N/A  Targeted Disease: HPV  Country/Currency (adj. year): Estonia;EUR (2016)  Cost estimates: Direct  Outcome measure: QALY | Modeling approach: Markov model  Time horizon: 88 years  Discounting: 5%  Sensitivity analysis: None  Threshold used: N/A | Results: €11,633–14,067 /QALY  Author's conclusions: Vaccination of 12-year-old girls alongside current cervical cancer screening can be considered a cost-effective intervention in Estonia with all three vaccines demonstrating similar ICERs.  % EE assessment satisfied: 89% |
| Willems (2021) | Perspective: Societal  Intervention/Comparator: Multi component intervention / General advice/ low intensity  Setting: School +  Targeted Disease: Type 2 diabetes mellitus  Country/Currency (adj. year): Other: Eupore;EUR (2016)  Cost estimates: Direct; Non-direct  Outcome measure: QALY | Modeling approach: Markov decision analytic model  Time horizon: 70 years  Discounting: Effects were discounted at 1.50% and costs at 3%  Sensitivity analysis: Deterministic; Probabilistic  Threshold used: Willingness-to-pay thresholds were based on the countries' gross domestic product per capita. | Results: The highest ICER was €12,033, corresponding to an incremental cost of €82,947 for 6.9 QALYs gained, per 1000 Finnish girls targeted.  Author's conclusions: Feel4Diabetes-intervention seems to be a low-cost intervention with small health gains on an individual level but substantial health gains on a population level, which is potentially cost-effective especially in those countries with a high overweight and obesity prevalence, and has a limited budget impact.  % EE assessment satisfied: 95% |
| Xu (2020) | Perspective: Societal  Intervention/Comparator: Physical activity and nutrition / Three comparators  Setting: Primary school  Targeted Disease: Obesity/Overweight  Country/Currency (adj. year): China;USD (2019)  Cost estimates: Other: N/A  Outcome measure: QALY | Modeling approach: Decision analytic model  Time horizon: 25 years  Discounting: 3%  Sensitivity analysis: Probabilistic  Threshold used: $50000/QALY | Results: 10,335.2 (1478.6)  Author's conclusions: Two single interventions were negative for the net benefit but all three school-based intervention measures for childhood obesity prevention were cost-effective, and economic analyses revealed that the comprehensive intervention was cost saving, and the cost per QALY saved was less than that of the other two interventions.  % EE assessment satisfied: 89% |
| Zandieh (2021) | Perspective: Healthcare  Intervention/Comparator: Supplements / No intervention  Setting: Secondary school  Targeted Disease: Cardiovascular diseases  Country/Currency (adj. year): Iran;USD (2018)  Cost estimates: Direct; Non-direct  Outcome measure: QALY | Modeling approach: Decision analytic model  Time horizon: 1 yr  Discounting: None  Sensitivity analysis: Probabilistic  Threshold used: 1032-2666 willingness-to-pay (WTP)/QALY | Results: 1090 USD per/QALY  Author's conclusions: Correcting the serum levels of vitamin D through a national vitamin D supplementation program offers substantial cost-saving to MOHME over a one-year time horizon to reduce the prevalence of MetS and help to CVD prevention.  % EE assessment satisfied: 89% |
| Zandieh (2022) | Perspective: Healthcare  Intervention/Comparator: Supplements / No intervention  Setting: Secondary school  Targeted Disease: Type 2 diabetes mellitus  Country/Currency (adj. year): Iran;USD (2018)  Cost estimates: Direct  Outcome measure: QALY | Modeling approach: Decision analytic model  Time horizon: 1 yr  Discounting: None  Sensitivity analysis: Probabilistic  Threshold used: 1032-2666 / QALY | Results: $4071.25 / QALY  Author's conclusions: Vitamin D supplementation for Iranian adolescents is highly cost-effective to reduce diabetes risk  % EE assessment satisfied: 84% |
| Zanganeh (2021) | Perspective: Societal  Intervention/Comparator: Multi component intervention / No intervention  Setting: Primary school  Targeted Disease: Obesity/Overweight  Country/Currency (adj. year): China;Yuan (2016/2017)  Cost estimates: Other: Unclear, but possibly both  Outcome measure: BMI | Modeling approach: Decision analytic model  Time horizon: 1 yr  Discounting: None  Sensitivity analysis: Probabilistic  Threshold used: UK (£20,000) and US (US$50,000) | Results: 8,888 Yuan (£1,760/US$2,502) / QALY from public perspective; 73,831 Yuan (£14,620/US$20,796) / QALY from societal perspective  Author's conclusions: From a public sector perspective and a societal perspective, this intervention is cost-effective and that this result was robust to all sensitivity analyses.  % EE assessment satisfied: % |
